# Children in Greenland: disease patterns and contacts to the health care system

**DOI:** 10.3402/ijch.v75.32903

**Published:** 2016-12-08

**Authors:** Marius Kløvgaard, Nina Odgaard Nielsen, Thomas Lund Sørensen, Peter Bjerregaard, Britta Olsen, Henrik Thybo Christesen

**Affiliations:** 1 Hans Christian Andersen Children’s Hospital, Odense University Hospital, Odense, Denmark; 2 Institute of Clinical Research, University of Southern Denmark, Odense, Denmark; 3 Regional Hospital, Ilulissat, Avannaa, Greenland; 4 National Institute of Public Health, Center for Health Research in Greenland, Copenhagen, Denmark; 5 Pediatric Department, Queen Ingrid’s Hospital, Nuuk, Greenland

**Keywords:** Greenland, health, paediatrics, disease, ICD-10, children, health care system, contacts

## Abstract

**Background:**

Previous studies of Greenlandic children’s disease pattern and contacts to the health care system are sparse and have focused on the primary health care sector.

**Objective:**

We aimed to identify the disease pattern and use of health care facilities of children aged 0–10 in two Greenlandic cohorts.

**Methods and design:**

In a retrospective, descriptive follow-up of the Ivaaq (The Greenland Child Cohort) and the CLEAR (climate changes, environmental contaminants and reproductive health) birth cohorts (total n=1,000), we reviewed medical records of children aged 6–10 in 2012 with residence in Nuuk or Ilulissat (n=332). Data on diseases and health care system contacts were extracted. Diagnoses were validated retrospectively. Primary health care contacts were reviewed for a random sample of 1:6.

**Results:**

In 311 children with valid social security number, the total number of health care system contacts was 12,471 equalling 4.6 contacts per child per year. The annual incidence rate of hospital admissions was 1:10 children (total n=266, 1,220 days, 4.6 days/admission), outpatient contacts 2:10 children and primary care 3.6 per child. Contacts were overall more frequent in boys compared with girls, 39.5 versus 34.6 during the study period, p=0.02. The highest annual contact rates for diseases were: hospitalisations/acute respiratory diseases 13.9:1,000; outpatient contacts/otitis media 5.1:1,000; primary care/conjunctivitis or nasopharyngitis 410:1,000 children. Outpatient screening for respiratory tuberculosis accounted 6.2:1,000, primary care non-disease (Z-diagnosis) 2,081:1,000 annually. Complete adherence to the child vaccination programme was seen in 40%, while 5% did not receive any vaccinations.

**Conclusions:**

In this first study of its kind, the health care contact pattern in Greenlandic children showed a relatively high hospitalisation rate and duration per admission, and a low primary health care contact rate. The overall contact rate and disease pattern resembled those in Denmark, except for tuberculosis screening. Adherence to the vaccination programme was low. These findings may be helpful for the organisation and dimensioning of the Greenlandic health care system for children.

Children comprise 26% of the population in Greenland (1), but little is known about the pattern of diseases and contacts to the health care system of this large proportion of the population. This information is important in organising and dimensioning the health care. In addition, rapid changes in living conditions and lifestyle may influence the patterns, calling for regular assessments. Some studies, though mostly at a smaller scale, have previously investigated some of these questions: in a study from 1991 including children in Nuuk, infectious diseases made up 66% of all contacts to the primary health care system (2). Among 350 Greenlandic children, who were referred to a paediatric outpatient clinic in eight Greenlandic districts in the period 1992–1994, the most frequent diseases reported were otitis media, epilepsy, asthma and atopic dermatitis (in the mentioned order) (3). Another study from 2010 to 2011 reported that among 25 boys and 25 girls aged 0–9, diseases of the respiratory tract, general and unspecific illnesses, and dermatological diseases made up the majority of contacts to the primary health care clinic in Nuuk, Greenland (4).

The contact pattern and the incidences of diseases among hospitalised children and for all health care contacts have, to the best of our knowledge not been described in Greenland before. Therefore, it is not known whether children in Greenland are more hospitalised or have different diseases than children in other populations. The aim of this study was to identify the entire disease pattern and the use of the health care facilities in two predefined cohorts of Greenlandic children up to 10 years of age, thereby making international comparison and future assessments possible.

## Methods

### Study setting and population

During the study period the health care system in Greenland consisted of the national hospital, Queen Ingrid’s Hospital, in the capital Nuuk, five regional hospitals, and health care stations in the smaller towns and settlements of the country. Furthermore, patients could be admitted to hospitals in Denmark and in acute situations to Iceland. Queen Ingrid’s Hospital served as the national hospital of Greenland as well as the local hospital in Nuuk. In addition, Nuuk had a primary health care clinic, which also served as the emergency room for the capital. All other hospitals in Greenland were considered as primary care facilities, but had wards where patients could be admitted.

The National Institute of Public Health, Center for Health Research in Greenland, Denmark, and The Primary Health Care Clinic, Nuuk, Greenland, established two mother-and-child cohorts in towns and settlements in Western Greenland, the Ivaaq cohort (The Greenland Child Cohort) (n=400) in 1999 and the CLEAR (climate changes, environmental contaminants and reproductive health) cohort (n=600) in 2002. The two cohorts included a sample of children born in Western Greenland from 1999 to 2005 and have been used for studies of pregnancy and birth (5–7). The ethnicity of the children in the cohorts is only partly known, but native Inuit have been mixed with Caucasians to a considerable degree. A recent study demonstrated that 27% of the Inuit genome in Greenland is of Scandinavian origin (8). Thus, this study examined the disease pattern at a geographic level, thereby using the term “Greenlandic children” instead of “Inuit children.”

### Identification of patients

**Fig. 1:**
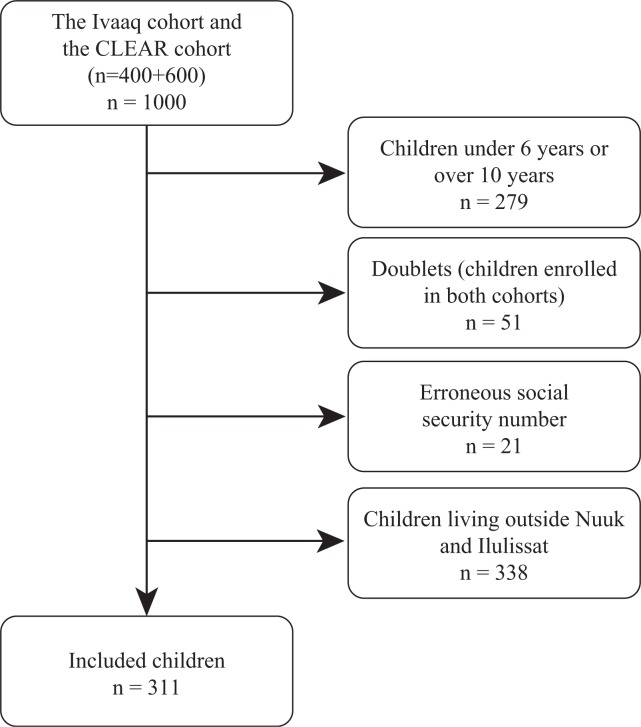
Flowchart for the study population, children aged 6–10 years in 2012.

From the Ivaaq cohort and the CLEAR cohort (n=1,000), we included children currently aged 6–10 in 2012 living in Nuuk or Ilulissat. Figure 1 shows the participant inclusion flowchart. The children were identified by date of birth with age determination at 15 October 2012. We retrospectively made a follow-up of the included children from birth to current age to examine the contact pattern to all health care sectors and the incidence of diseases among Greenlandic children.

### Data collection

Medical records in all electronic archives and databases at all hospitals and health care stations in Greenland and at the primary health care clinic in Nuuk were identified. To complete the data collection, all medical records in the physical archives in Nuuk and Ilulissat were included. In addition, hospitalisations in Denmark for specialist care were identified from the medical records due to discharge summaries from the Danish hospitals. All contacts to the health care system from birth up to 15 October 2012 were included. Contacts to the primary health care clinic in Nuuk as well as contacts to the local physicians at the regional hospitals were regarded as contacts to the primary health care sector. Outpatient contacts to the specialists at Queen Ingrid’s Hospital or to visiting specialists, for example, paediatricians, otolaryngologists and ophthalmologists, at the regional hospitals were regarded as contacts to the secondary health care sector. All hospitalisations at either Queen Ingrid’s Hospital, the regional hospitals or hospitals in Denmark were also regarded as contacts to the secondary health care sector. Contacts to physicians or nurses in the primary health care sector were included, because nurses at the Greenlandic hospitals often treat, by delegation from a physician, a broad sample of diseases. If a contact to a nurse was followed by a contact to a physician the same or the following day, only the latter was included. Telephone consultations and email consultations were also included. If a telephone or email consultation was followed by a physical consultation on the same or the following day, only the physical consultation was included. If a child had more than one contact to the primary health care sector on the same day, only one of the contacts was included. Contacts about renewal or change of medical prescription, and contacts or consultations about fatherhood, and mail correspondences without disease consultation content were excluded.

Hospitalisations in either Greenland or Denmark were indexed by the date of admission, the date of discharge, the total days of admission and the diagnosis by the WHO ICD-10 classification system (9). Outpatient consultations within the secondary health care sector were indexed by the date of the contact and the diagnosis by the ICD-10 classification system. Contacts within the primary health care sector were indexed by the date of the contact and whether the contact was due to illness, the Greenlandic child examination programme or the Greenlandic child vaccination programme. For vaccinations, children who had received all vaccinations were categorised as complete adherence, children who had received one vaccination but not all were classified as incomplete adherence and children who had no vaccinations were categorised as no adherence. Furthermore, in a random sample of 52 children (every sixth listed child in the study population), each contact to the primary health care sector was indexed with an ICD-10 diagnosis code as described for all contacts to the secondary health care sector.

Contacts to child psychiatrists were not available from the archives or databases and were not a part of the study. Normal births without neonatal complications were not included in our study.

The indexation by the ICD-10 codes was primarily based on the ICD-10 code used by the physician in the medical record, but validated for each contact by the first author by medical file review.

In the case of a child with more contacts due to the same disease, follow-up contacts were retrospectively indexed with a follow-up code to avoid double-registration of diseases, while all contacts for the same disease were counted for the contact number evaluation. For example, two contacts due to the same otitis media, for example, within 1 week, would count as one otitis media in the incidence evaluation, but as two contacts in the frequency of contacts evaluation.

### Data analysis and statistics

Data were anonymised. Contacts were counted according to ICD-10 codes and grouped into primary categories of the ICD-10 classification system (9) to ease comparisons between disease groups. Incidence rates for both individual diagnoses and disease categories were calculated. For descriptive statistics, differences between groups were assessed by Pearson’s chi-square test or independent *t*-test. Differences between means within the same group were assessed by paired *t*-test using SPSS. The level of statistical significance was p<0.05.

### Ethics

The study complied with the Helsinki declaration and was approved by the Greenlandic Committee on Health Research Ethics (Ref.no. 2012-069391), the Danish Data Protection Agency (J.no. 2012-41-0735) and the Agency for Health and Prevention in Greenland.

## Results

We identified 311 (96.7%) of the eligible 332 children from the Ivaaq and the CLEAR cohorts. Of these, 198 children lived in Nuuk (104 boys and 94 girls) and 113 in Ilulissat (61 boys and 52 girls). The mean age in the study population was 8.8 years by 15 October 2012. The mortality during the study period was zero. The study population made up 7% of the 4,453 Greenlandic children born in the period 2002–2006 (10).

Table I shows the pattern of contacts to the Greenlandic health care system for the study population. The children had a total of 12,471 contacts to the Greenlandic health care sector, equal to 4.6 annual contacts per child, including contacts due to diseases and the Greenlandic child examination or vaccination programmes. Of these, 4.2 annual contacts per child were in the primary health care sector. Boys had more contacts to the primary health care sector compared with girls, mean 39.5 versus 34.6 contacts during the study period, p=0.02.

**Table I:** Contacts to the Greenlandic health care system grouped by place of living and child sex

	Hospital admissions		Outpatient contacts to the secondary health care sector		Contacts to the primary health care sector		Contacts to the primary health care sector; mandatory child examinations and vaccinations excluded		Total of contacts (admissions, outpatients and primary)		Total of contacts (admissions, outpatients and primary); mandatory child examinations and vaccinations excluded	
						
	N	Mean (SD)	N	Mean (SD)	N	Mean (SD)	N	Mean (SD)	N	Mean (SD)	N	Mean (SD)
Nuuk												
Male	77	0.7 (±1.1)	212	2.0 (±3.2)	3,985	38.3 (±18.6)	3,244	31.2 (±17.7)	4,274	41.1 (±20.4)	3,533	34.0 (±19.5)
Female	65	0.7 (±1.1)	215	2.3 (±3.9)	3,299	35.1 (±16.6)	2,680	28.5 (±16.2)	3,579	38.1 (±18.2)	2,960	31.5 (±17.8)
Total	142	0.7 (±1.1)	427	2.2 (±3.6)	7,284	36.8 (±17.7)	5,924	29.9 (±17.0)	7,853	39.7 (±19.4)	6,493	32.8 (18.7)
Ilulissat												
Male	93	1.5 (±3.6)	149	2.4 (±4.5)	2,536	41.6 (±22.4)	2,130	34.9 (±21.8)	2,778	45.5 (±27.1)	2,372	38.9 (±26.4)
Female	31	0.6 (±1.1)	63	1.2 (±3.3)	1,746	33.6 (±15.4)	1,389	26.7 (±14.8)	1,840	35.4 (17.2)	1,483	28.5 (±16.7)
Total	124	1.1 (±2.8)	212	1.9 (±4.0)	4,282	37.9 (±19.8)	3,519	31.1 (±19.3)	4,618	40.9 (±23.6)	3,855	34.1 (±23.0)
Nuuk+Ilulissat												
Male total	170	1.0 (±2.4)	361	2.2 (±3.8)	6,521	39.5 (±20.1)	5,374	32.6 (±19.3)	7,052	42.7 (±23.1)	5,905	35.8 (±22.3)
Female total	96	0.7 (±1.1)	278	1.9 (±3.7)	5,045	34.6 (±16.2)	4,069	27.9 (±15.7)	5,419	37.1 (±17.8)	4,443	30.4 (±17.4)
Total	266	0.9 (±1.9)	639	2.1 (±3.7)	11,566	37.1 (±18.5)	9,443	30.4 (±17.9)	12,471	40.1 (±21.0)	10,348	33.3 (±20.3)

N=number of contacts; SD=standard deviation.

**Fig. 2:**
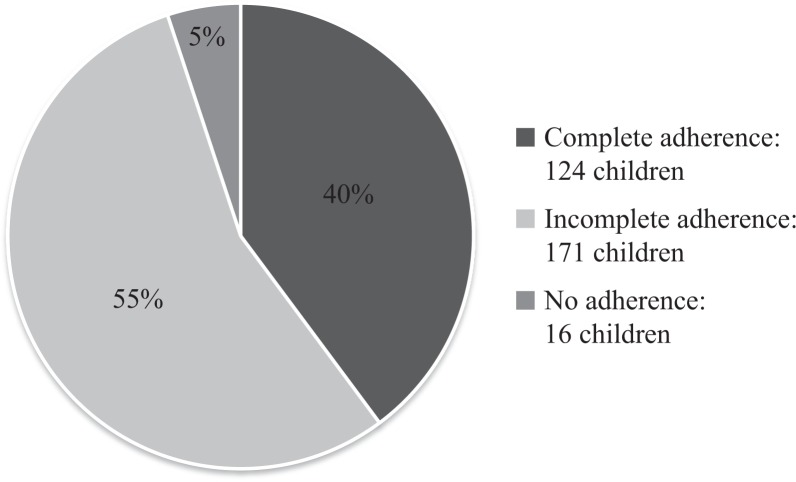
Adherence to the Greenlandic child vaccination programme.

The Greenlandic child vaccination programme consisted of six vaccinations up to the age of 10 (11). Figure 2 shows the adherence to the vaccination programme grouped into complete adherence, incomplete adherence and no adherence. Forty percent of the children had a complete adherence, while 55% were incomplete. Five percent did not follow the programme at all.

For disease contacts only, 10,348 contacts to the health care system were recorded, equalling 3.8 annual contacts per child. Of these, 266 contacts were hospital admissions with a total of 1,220 hospital days (including 35 hospitalisations in Denmark with a total of 295 hospital days), giving a mean of 4.6 hospital days per hospitalisation (mean of 8.4 hospital days for hospitalisations in Denmark). The number of hospitalisations per child ranged from 0 (n=180) to 21 (n=1), as shown in Supplementary Table I. The annual hospitalisation incidence rate was 1 hospitalisation per 10 children. Outpatient contacts to the secondary health care sector numbered 639 (mean 2.1 per child). The annual outpatient incidence rate was 2 per 10 children.

Contacts to the primary health care sector due to illnesses numbered 9,443 (mean 30.4 per child). The annual primary care contact rate was 3.5 per child due to illnesses.

A trend towards increased hospitalisation rate was found in Nuuk compared with Ilulissat (both genders, p=0.09). No differences were seen for outpatient consultations or contacts to the primary health care sector between the two towns (p=0.5 and p=0.6, respectively). Furthermore, no difference was seen in the length of hospitals admission between children from Nuuk and Illulissat (mean of 4.5 and 4.7 hospital days per hospitalisation, respectively, p=0.8).

The children had most contacts to the health care sector during their first years of living, as shown in Supplementary Table II. For example, 48% of all hospitalisations and 45% of all contacts to the primary health care sector were of children up to 2 years. Yet, outpatient contacts were more equally distributed with a mean number of contacts per year between 0.1 and 0.3 from birth to 10 years of age.

### Diseases by hospitalisation

The 10 most frequent diseases and disease groups recorded for hospital admissions and their incidence rates are shown in Table II. Pneumonia, acute bronchiolitis and febrile convulsions accounted for the three most frequent diseases with an annual incidence rate of 8.4, 5.5 and 5.5 per 1,000 children, respectively.

**Table II:** The incidence rate of the 10 most frequent diseases and disease groups for hospital admissions

	N	Mean (SD)	Incidence rate (1,000/year)
ICD-10 code			
Pneumonia UNS (J189)	23	0.07 (±0.30)	8.4
Observation for suspected disease or condition UNS (Z039)	20	0.06 (±0.27)	7.3
Acute bronchitis UNS (J209)	15	0.05 (±0.24)	5.5
Febrile convulsions (R560)	15	0.05 (±0.43)	5.5
Follow-up examination after medical treatment (Z095)	15	0.05 (±0.42)	5.5
Concussion (S060)	11	0.04 (±0.19)	4.0
Unilateral or unspecified inguinal hernia, without obstruction or gangrene (K409)	10	0.03 (±0.33)	3.7
Hypertrophy of adenoids (J352)	8	0.03 (±0.16)	2.9
Viral infection UNS (B349)	8	0.03 (±0.18)	2.9
Chronic mucoid otitis media (H65.2)	7	0.02 (±017)	2.6
ICD-10 category			
Diseases of the respiratory system [DJ00-DJ99]	73	0.23 (±0.68)	26.7
Symptoms, signs and abnormal clinical and laboratory findings, not elsewhere classified [DR00-DR99]	45	0.14 (±0.78)	16.4
Factors influencing health status and contact with health services [DZ00-DZ99]	40	0.13 (±0.59)	14.7
Diseases of the ear and mastoid process [DH60-DH95]	35	0.11 (±0.70)	12.8
Injury, poisoning and certain other consequences of external causes [DS00-DT98]	34	0.11 (±0.36)	12.4
Certain infectious and parasitic diseases [DA00-DB99]	25	0.08 (±0.30)	9.1
Diseases of the digestive system [DK00-DK93]	21	0.07 (±0.40)	7.7
Congenital malformations, deformations and chromosomal abnormalities [DQ00-DQ99]	19	0.06 (±0.30)	6.9
Diseases of the nervous system [DG00-DG99]	18	0.06 (±0.44)	6.6
Certain conditions originating in the perinatal period [DP00-DP96]	15	0.05 (±0.30)	5.5

N=number of contacts; SD=standard deviation.

Grouped into the primary categories, diseases of the respiratory system had by far the highest incidence rate of 26.7 per 1,000 per year. For both individual disease codes and disease groups, unspecified diagnoses were prevalent even after re-evaluation of the medical files.

Supplementary Tables III and IV show the five most frequent diseases and disease groups for hospitalisations for boys and girls and Nuuk and Ilulissat. Among the five most frequent diseases, follow-up examination was found in boys, whereas prematurity and unclassified fever were only found in girls. Pneumonia and diseases of the respiratory system were the most frequent diagnosis or disease group in both child sexes.

### Diseases by outpatient contacts

A high percentage of the outpatient consultations in the secondary health care sector were due to either follow-up examinations of already diagnosed diseases, or observations for suspected diseases or conditions, or where no relevant diagnosis could be found in the medical record. Apart from these contacts, screening for respiratory tuberculosis, otitis media and hypermetropia were the most frequent contact diagnoses with an annual incidence rate of 6.2, 5.1 and 4.8 per 1,000, respectively, Table III. The most frequent disease groups were diseases of the eye, examinations due to symptoms and signs, and diseases of the ear with an incidence rate of 18.7, 16.5 and 14.3 per 1,000 per year, respectively.

**Table III:** The incidence rate for the 10 most frequent diseases and disease groups for outpatient consultations in the secondary sector

	N	Mean (SD)	Incidence rate (1,000/year)
ICD-10 code			
Special screening for respiratory tuberculosis (Z111)	17	0.05 (±0.25)	6.2
Otitis media UNS (H669)	14	0.05 (±0.26)	5.1
Hypermetropia (H520)	13	0.04 (±0.20)	4.8
Hypertrophy of adenoids (J352)	9	0.03 (±0.17)	3.3
Hypertrophy of tonsils (J351)	8	0.03 (±0.16)	2.9
Epistaxis (R040)	8	0.03 (±0.18)	2.9
Perforation of tympanic membrane UNS (H729)	7	0.02 (±0.15)	2.6
Atopic dermatitis UNS (L209)	7	0.02 (±0.15)	2.6
Constipation (K590)	7	0.02 (±0.17)	2.6
Astigmatism (H522)	6	0.02 (±0.14)	2.2
ICD-10 category			
Diseases of the eye and adnexa [DH00-DH59]	51	0.16 (±0.59)	18.7
Symptoms, signs and abnormal clinical and laboratory findings, not elsewhere classified [DR00-DR99]	45	0.14 (±0.47)	16.5
Diseases of the ear and mastoid process [DH60-DH95]	39	0.13 (±0.51)	14.3
Diseases of the respiratory system [DJ00-DJ99]	29	0.09 (±0.35)	10.6
Congenital malformations, deformations and chromosomal abnormalities [DQ00-DQ99]	22	0.07 (±0.37)	8.0
Diseases of the skin and subcutaneous tissue [DL00-DL99]	17	0.05 (±0.31)	6.2
Diseases of the digestive system [DK00-DK93]	15	0.05 (±0.24)	5.5
Mental and behavioural disorders [DF00-DF99]	9	0.03 (±0.22)	3.3
Diseases of the musculoskeletal system and connective tissue [DM00-DM99]	7	0.02 (±0.17)	2.6
Certain infectious and parasitic diseases [DA00-DB99]	7	0.02 (±0.17)	2.6

N=number of contacts; SD=standard deviation. Z0 diagnosis codes and Z-category excluded.

Supplementary Tables V and VI show the five most frequent diseases and disease groups for outpatient contacts for boys and girls and Nuuk and Ilulissat. Among the five most frequent diseases for outpatient contacts, screening for tuberculosis was only found in Nuuk and in girls, while otitis media was only found in Nuuk and in boys. In both Nuuk and Ilulissat, diseases of the eye were among the most frequent disease groups. In girls, diseases of the eye were the most frequent disease group, while diseases of the ears were most common among boys.

### Diseases by primary health care contacts

For a random sample of 52 children (one-sixth of the study population, mean age 8.4 years at follow-up), all contacts to the primary health care sector were indexed by ICD-10 codes and incidence rates were calculated. The sample had a total of 1,574 disease contacts to the primary health care sector, equalling 3.6 annual contacts per child.

Table IV shows the 10 most frequent diseases and disease groups for the primary health care sector and their incidence rates. As for the outpatient consultations, a high percentage of the contacts to the primary health care sector were due to observation for suspected diseases or follow-up examinations. The incidence rate for consultations due to the ICD-10 category Z (e.g. investigations, observations for diseases, screenings and vaccinations) was 2,081.0 per 1,000 per year. Apart from these contacts, conjunctivitis, nasopharyngitis, rash and otitis media were the most frequent diseases with an incidence rate of 210.6, 199.2, 169.4 and 167.1 per 1,000 per year, respectively. The most frequent disease groups, Z-diagnoses excluded, were examinations due to symptoms and signs, respiratory diseases and diseases of the ear with incidence rates of 636.4, 590.7 and 341.1 per 1,000 per year, respectively.

**Table IV:** The incidence rate for the 10 most frequent diseases and disease groups in the primary sector

	N	Mean (SD)	Incidence rate (1,000/year)
ICD-10 code			
Conjunctivitis UNS (H109)	92	1.77 (±2.24)	210.6
Acute nasopharyngitis UNS (J009)	87	1.67 (±1.84)	199.2
Rash UNS (R219)	74	1.42 (±1.66)	169.4
Otitis media UNS (H669)	73	1.40 (±1.77)	167.1
Cough UNS (R059)	68	1.31 (±1.37)	155.7
Fever UNS (R509)	67	1.29 (±1.76)	153.4
Acute bronchitis UNS (J209)	57	1.10 (±1.24)	130.5
Impetigo UNS (L010)	45	0.87 (±1.37)	103.0
Pneumonia UNS (J189)	37	0.71 (±1.04)	84.7
Acute tonsillitis UNS (J039)	33	0.63 (±0.93)	75.5
ICD-10 category			
Factors influencing health status and contact with health services [DZ00-DZ99]	909	17.48 (±7.97)	2,081.0
Symptoms, signs and abnormal clinical and laboratory findings, not elsewhere classified [DR00-DR99]	278	5.35 (±3.40)	636.4
Diseases of the respiratory system [DJ00-DJ99]	258	4.96 (±3.78)	590.7
Diseases of the ear and mastoid process [DH60-DH95]	149	2.87 (±2.64)	341.1
Certain infectious and parasitic diseases [DA00-DB99]	143	2.75 (±2.34)	327.4
Injury, poisoning and certain other consequences of external causes [DS00-DT98]	106	2.04 (±1.30)	242.7
Diseases of the eye and adnexa [DH00-DH59]	104	2.00 (±2.25)	238.1
Diseases of the skin and subcutaneous tissue [DL00-DL99]	102	1.96 (±2.11)	233.5
Diseases of the digestive system [DK00-DK93]	39	0.75 (±0.81)	89.3
Diseases of the genitourinary system [DN00-DN99]	15	0.29 (±0.61)	34.3

N=number of contacts; SD=standard deviation. Study population=52. Z0 diagnosis codes excluded.

Supplementary Tables VII and VIII show the five most frequent diseases and disease groups for the primary health care sector for boys and girls and Nuuk and Ilulissat. Among the five most frequent diseases, conjunctivitis was the most common among both sexes, while otitis media was only found in boys and unspecified fever was only found in girls. Respiratory diseases and examinations due to symptoms and signs were the most frequent disease groups in both child sexes as well as in both towns. No diagnoses were found for battered child, sexual abuse or neglect.

## Discussion

Our follow-up of 311 children aged up to 10 years from the capital Nuuk and Ilulissat, one of the bigger towns in Greenland, showed 4.6 health care contacts per child per year, by far mostly in the primary health care sector and during the first years of living. There were no significant differences in the contact patterns between Nuuk and Ilulissat. Primary care contacts without actual disease were most frequent, and outpatient screening for respiratory tuberculosis were more frequent than for any specific disease. Dominating diseases were respiratory infections and bronchitis for hospitalisations, otitis media for secondary health care contacts and conjunctivitis and nasopharyngitis for primary care contacts. Forty percent of the children had a complete adherence to the Greenlandic vaccination programme, while 5% did not attend the programme at all.

Yet, a very small group of children with severe handicap born in Nuuk or Ilulissat were no longer living in these two towns because they had been moved to institutions for special care. These children with a high frequency of health care contacts were not included in our study.

Furthermore, the study population represented a fraction of Greenlandic children living in towns. Children living in settlements might have another contact patterns to the health care system due to the variation in health care facilities throughout Greenland.

### Contacts to the health care system

The overall pattern of contacts to the health care system in Greenland has never been analysed before, and to the best of our knowledge, no studies about contacts to the health care sector in other Inuit communities, for example, in Canada or Alaska, are to be found either.

Data from Denmark (12) showed that Danish children during a similar period on average had an annual hospital rate of 0.6 per 10 children with an average duration of 3.3 days per admission, and 5.5 annual contacts to the primary health care sector per child. Compared with Denmark, the Greenlandic children had on average 67% extra hospitalisations and on average 1.3 extra hospitalisation days per admission. On the contrary, the Greenlandic children had 24% fewer contacts to the primary health care sector.

The increased number and duration of hospital admissions may be due to the organisation of the health care sector. In Greenland, patients living in other towns than Nuuk must travel long distances to be treated by specialists. Patients might even be hospitalised for outpatient treatment with the risk of prolonged admission until the return transport is possible, unless the patient can wait until a specialist visits the patient’s hometown, which may happen on a yearly basis. Yet, no difference was seen in the average hospitalisation days between children in Nuuk and Ilulissat. Seventeen children (Nuuk 9 and Ilulissat 8) were hospitalised 35 times in Denmark for specialist treatment, in approximately 40% of the cases due to congenital and genetic disorders (Q-diagnoses). The 35 hospitalisations in Denmark accounted for approximately 10% of all hospitalisations and were of longer duration, that is, 25% of all hospitalisation days were in Denmark. Again, this might be because the patient had to wait for the return transportation to be possible. Furthermore, the health care system in Greenland only employed two specialists in paediatrics during the study period as well as visiting consultants from Denmark, which made it possible to see a paediatrician in all towns and settlements once a year, and twice a year in Nuuk. In Greenland, the primary health care sector is physically located at the local hospitals and health care stations and is employed by doctors, who also work at the hospitals. This way of organising the health care sector might make it easier for physicians to admit children to the hospital and thereby increasing the hospital admission rate. Yet, this study did not find a higher hospitalisation rate in Ilulissat compared with Nuuk. Another reason for a higher frequency of hospital admissions and longer admissions could be that children in Greenland have more severe diseases. However, this was not the finding of this study.

The adherence to the Greenlandic child vaccination programme was not complete. A study from Denmark from 2004 found that 91–93% of the Danish children followed the child vaccination programme during the first 15 month and that the adherence fell to 81% after 5 years (13). Compared with Denmark, the adherence to the vaccination programme in Greenland seems lower. Yet, the Danish study analysed the adherence to each vaccination and not the proportion of children who had received all vaccinations in the programme.

### Incidence of diseases

The causes of hospital admissions among children in Greenland have to the best of our knowledge never been analysed before. The three most common ICD-10 codes for hospital admissions were pneumonia, observation for suspected disease or condition and acute bronchitis. In Denmark, the most common causes for hospital admissions were from categories of other diseases and symptoms, and diseases of the respiratory system (12), while a Danish survey from 2005 found that the most common acute as well as chronic disease group among children was respiratory diseases (14). In both countries, respiratory diseases were among the most frequent causes for hospital admissions. No systematic studies of incidence rates of health care contacts by diseases for Danish children, nor from Inuit children in Canada or Alaska, could be found for comparison. However, studies of hospitalisations due to respiratory diseases among Inuit children in parts of Canada have found the hospitalisation rate for bronchiolitis to be up to 306 per 1,000 per year during the first year of life (15,16). In comparison, this study found a hospitalisation incidence rate due to acute respiratory diseases of 13.9 per 1,000 per year.

As for outpatient consultation only, Becker-Christensen (3) found the most common causes for outpatient consultations to a paediatric clinic in 1992–1994 (Z-diagnoses excluded) to be otitis media, epilepsy, asthma and atopic dermatitis. Grouped into the primary ICD-10 categories, the most common causes for outpatient contacts were diseases of the respiratory system, symptoms, signs and abnormal clinical and laboratory findings, not elsewhere classified, and diseases of the nervous system. Compared with Becker-Christensen, our study found a higher incidence of eye diseases especially hypermetropia, which is known to have a higher incidence among Inuit compared with other ethnic groups (17). One reason for this finding could therefore be our inclusion of all outpatient consultations, thereby also contacts to ophthalmologists, and not only paediatric contacts as in the Becker-Christensen study. Furthermore, the consultations examined by Becker-Christensen might not reflect incidence of diseases in a community, but merely referral patterns to the specialised paediatric function.

As for contacts to the primary health care sector, Pedersen et al. (4) examined 50 children aged 0–9 with contacts to the primary health care clinic in Nuuk in 2010–2011. No incidence rates were reported. The most common causes for contacts were respiratory diseases, general and unspecified diseases, skin diseases, diseases of the ear and diseases of the eye. The same pattern was found in our study, in both Nuuk and Ilulissat.

Others have previously found a high incidence of otitis media among children in Greenland (18,19), which was also found in this study. The incidence of otitis media was 167.1 and 5.1 per 1,000 per year in the primary health care sector and in the outpatient clinics, respectively. However, among patients in the outpatient clinics, diseases of the eye were more frequent than diseases of the ear.

We did not find any contacts to the Greenlandic health care sector due to battered child, sexual abuse or neglect. The latter is somewhat in contrast to recently published population surveys from Northern Greenland, where 39.5% of all women and 16.9% of all men had experienced sexual abuse during childhood (20). Earlier studies from Greenland found that 28% of girls and 9% of boys had experienced sexual abuse (21). Potential explanations to this discrepancy include the maximal age of 10 years in our study, selection bias in the cohort sample compared with the population background, lack of contacts to the health care system in these matters or underdiagnosing where contacts could have suggested a problem. The null findings may therefore not reflect the actual rates of sexual abuse, violence and neglect, even though public employees in Greenland have increased obligations of notification on suspicion (22).

## Strengths and limitations

Strengths of our study included the population-based design, the inclusion of data from all health care levels and the relatively large study population.

Limitations included the retrospective nature of our study with risk of report bias. The medical records of the secondary health care sector included for most contacts an ICD-10 diagnosis code. This was not the case for the medical records in the primary health care sector. Therefore, a lot of diagnoses had to be given based on the information in the medical records. Given the sometimes paucity of data, this may have led to a higher occurrence of unspecified diagnosis and use of the ICD-10 category symptoms, signs and abnormal clinical and laboratory findings, not elsewhere classified. Another reason could be that the children attended a physician without being sick. Yet, the ICD-10 classification system was used to make comparison with other studies possible.

Missing data could furthermore not be excluded, as the Greenlandic medical records were transformed from printed archives to electronic medical records during the study period, which may not have been processed in completeness.

Lastly, our findings are based on data from Nuuk and Ilulissat, which may not be representative for other towns or settlements in Greenland, including adherence to the vaccination programme.

## Conclusion

Children in Greenland were more often admitted to a hospital and had longer hospital admissions, but fewer contacts to the primary health care sector compared with Denmark. Outpatient screening for respiratory tuberculosis was frequent. The adherence rate to the vaccination programme was low. Respiratory diseases, diseases of the eyes and ears, and symptoms and abnormal findings were common causes of contacts to the health care system. The absence of diagnoses for battered child, neglect and sexual abuse may not reflect the real occurrence of these problems.

Our findings may be helpful for the organisation and dimensioning of the Greenlandic health care system for children.

## Supplementary Material

Children in Greenland: disease patterns and contacts to the health care system
