# Supplementary material for: Children in Greenland: disease patterns and contacts to the health care system
Source: Int J Circumpolar Health. 2016 Dec 8;75:10.3402/ijch.v75.32903. doi: 10.3402/ijch.v75.32903 (PMC5148806; doi:10.3402/ijch.v75.32903)
Supplement: Children in Greenland: disease patterns and contacts to the health care system [file IJCH-75-32903-s001.pdf]

Supplementary tables

Supplementary Table 1: Frequency of hospital admissions among children.

Number of hospital admissions	Number of children	Percentage
0	180	57.9%
1	80	25.7%
2	25	8.0%
3	10	3.2%
4	8	2.6%
5	3	1.0%
6	2	0.6%
10	1	0.3%
16	1	0.3%
21	1	0.3%
**Total**	**311**	**100.0%**

Supplementary Table 2: The five most frequent ICD-10 codes for hospital admissions for boys/girls and Nuuk/Ilulissat.

	ICD-10 code	N	Mean (SD)	Incidence rate (1000/year)
**Boys**				
	Observation for suspected disease or condition UNS (Z039)	14	0.08 (±0.32)	9.6
	Pneumonia UNS (J189)	12	0.07 (±0.32)	8.3
	Epilepsy UNS (G409)	10	0.06 (±0.51)	6.9
	Febrile convulsions (R560)	10	0.06 (±0.56)	6.9
	Acute bronchitis UNS (J209)	10	0.06 (±0.29)	6.9
**Girls**				
	Pneumonia UNS (J189)	11	0.08 (±0.26)	8.7
	Observation for suspected disease or condition UNS (Z039)	6	0.04 (±0.20)	4.7
	Febrile convulsions (R560)	5	0.03 (±0.18)	3.9
	Acute bronchitis UNS (J209)	5	0.03 (±0.18)	3.9
	Prematurity (P073)	4	0.03 (±0.26)	3.2
	Fever UNS (R509)	4	0.03 (±0.16)	3.2
**Nuuk**				
	Pneumonia UNS (J189)	11	0.06 (±0.23)	6.2
	Observation for suspected disease or condition UNS (Z039)	10	0.05 (±0.22)	5.7
	Unilateral or unspecified inguinal hernia, without obstruction or gangrene (K409)	9	0.05 (±0.41)	5.1
	Concussion (S060)	8	0.04 (±0.20)	4.5
	Acute bronchitis UNS (J209)	8	0.04 (±0.22)	4.5
**Ilulissat**				
	Pneumonia UNS (J189)	12	0.11 (±0.39)	12.5
	Febrile convulsions (R560)	11	0.10 (±0.68)	11.4
	Observation for suspected disease or condition UNS (Z039)	10	0.09 (±0.34)	10.4
	Epilepsy UNS (G409)	8	0.07 (±0.59)	8.3
	Follow-up examination after medical treatment (Z095)	8	0.07 (±0.44)	8.3

N=number of contacts, SD=standard deviation.

Supplementary Table 3: The five most frequent ICD-10 categories for hospital admissions for boys/girls and Nuuk/Ilulissat.

	ICD-10 category	N	Mean (SD)	Incidence rate (1000/ year)
**Boys**				
	Diseases of the respiratory system [DJ00-DJ99]	46	0.28 (±0.84)	31.6
	Symptoms, signs and abnormal clinical and laboratory findings, not elsewhere classified [DR00-DR99]	29	0.18 (±0.98)	19.9
	Diseases of the ear and mastoid process [DH60-DH95]	25	0.15 (±0.90)	17.2
	Factors influencing health status and contact with health services [DZ00-DZ99]	25	0.15 (±0.54)	17.2
	Injury, poisoning and certain other consequences of external causes [DS00-DT98]	25	0.15 (±0.39)	17.2
**Girls**				
	Diseases of the respiratory system [DJ00-DJ99]	27	0.18 (±0.44)	21.3
	Symptoms, signs and abnormal clinical and laboratory findings, not elsewhere classified [DR00-DR99]	16	0.11 (±0.44)	12.6
	Certain infectious and parasitic diseases [DA00-DB99]	12	0.08 (±0.32)	9.5
	Certain conditions originating in the perinatal period [DP00-DP96]	10	0.07 (±0.40)	7.9
	Diseases of the ear and mastoid process [DH60-DH95]	10	0.07 (±0.36)	7.9
**Nuuk**				
	Diseases of the respiratory system [DJ00-DJ99]	48	0.24 (±0.55)	27.2
	Injury, poisoning and certain other consequences of external causes [DS00-DT98]	20	0.10 (±0.35)	11.4
	Diseases of the ear and mastoid process [DH60-DH95]	20	0.10 (±0.39)	11.4
	Symptoms, signs and abnormal clinical and laboratory findings, not elsewhere classified [DR00-DR99]	16	0.08 (±0.31)	9.1
	Diseases of the digestive system [DK00-DK93]	16	0.08 (±0.47)	9.1
**Ilulissat**				
	Symptoms, signs and abnormal clinical and laboratory findings, not elsewhere classified [DR00-DR99]	29	0.26 (±1.22)	30.1
	Diseases of the respiratory system [DJ00-DJ99]	25	0.22 (±0.86)	26.0
	Factors influencing health status and contact with health services [DZ00-DZ99]	19	0.17 (±0.61)	19.7
	Diseases of the nervous system [DG00-DG99]	18	0.16 (±1.03)	18.7
	Diseases of the ear and mastoid process [DH60-DH95]	15	0.13 (±1.05)	15.6

N=number of contacts, SD=standard deviation.

Supplementary Table 4: The five most frequent ICD-10 codes for outpatient consultations for boys/girls and Nuuk/Ilulissat.

ICD-10 code	N	Mean (SD)	Incidence rate (1000/year)
**Male**			
Otitis media UNS (H669)	13	0.08 (±0.35)	8.9
Hypertrophy of adenoids (J352)	10	0.06 (±0.33)	6.9
Hypermetropia (H520)	7	0.04 (±0.23)	4.8
Hypertrophy of tonsils (J351)	7	0.04 (±0.23)	4.8
Special screening for respiratory tuberculosis (Z111)	6	0.04 (±0.22)	4.1
Delayed milestone (R620B)	6	0.04 (±0.24)	4.1
Epistaxis (R040)	6	0.04 (±0.22)	4.1
Perforation of tympanic membrane UNS (H729)	6	0.04 (±0.19)	4.1
**Female**			
Special screening for respiratory tuberculosis (Z111)	11	0.08 (±0.29)	8.7
Hypermetropia (H520)	7	0.05 (±0.21)	5.5
Astigmatism (H522)	6	0.04 (±0.20)	4.7
Atopic dermatitis UNS (L209)	5	0.03 (±0.18)	3.9
Constipation (K590)	5	0.03 (±0.22)	3.9
**Nuuk**			
Special screening for respiratory tuberculosis (Z111)	17	0.09 (±0.32)	9.7
Otitis media UNS (H669)	12	0.06 (±0.31)	6.8
Hypertrophy of adenoids (J352)	9	0.05 (±0.29)	5.1
Hypertrophy of tonsils (J351)	8	0.04 (±0.24)	4.5
Constipation (K590)	7	0.04 (±0.21)	4.0
**Ilulissat**			
Hypermetropia (H520)	8	0.07 (±0.29)	8.3
Atopic dermatitis UNS (L209)	6	0.05 (±0.23)	6.2
Esotropia alternating (H500A)	5	0.04 (±0.28)	5.2
Hypertrophy of adenoids (J352)	3	0.03 (±0.16)	3.1
Perforation of tympanic membrane UNS (H729)	3	0.03 (±0.16)	3.1

N=number of contacts, SD=standard deviation.
Z0-diagnoses excluded.

Supplementary Table 5: The five most frequent ICD-10 categories for outpatient consultations for boys/girls and Nuuk/Ilulissat.

	ICD-10 category	N	Mean (SD)	Incidence rate (1000/year)
**Male**				
	Diseases of the ear and mastoid process [DH60-DH95]	35	0.21 (±0.67)	24.1
	Symptoms, signs and abnormal clinical and laboratory findings, not elsewhere classified [DR00-DR99]	34	0.21 (±0.63)	23.4
	Diseases of the respiratory system [DJ00-DJ99]	23	0.14 (±0.47)	15.8
	Diseases of the eye and adnexa [DH00-DH59]	22	0.13 (±0.63)	15.1
	Congenital malformations, deformations and chromosomal abnormalities [DQ00-DQ99]	13	0.08 (±0.40)	8.9
**Female**				
	Diseases of the eye and adnexa [DH00-DH59]	32	0.22 (±0.68)	25.2
	Symptoms, signs and abnormal clinical and laboratory findings, not elsewhere classified [DR00-DR99]	13	0.09 (±0.31)	10.2
	Diseases of the respiratory system [DJ00-DJ99]	11	0.08 (±0.37)	8.7
	Congenital malformations, deformations and chromosomal abnormalities [DQ00-DQ99]	9	0.06 (±0.34)	7.1
	Diseases of the skin and subcutaneous tissue [DL00-DL99]	8	0.05 (±0.23)	6.3
**Nuuk**				
	Symptoms, signs and abnormal clinical and laboratory findings, not elsewhere classified [DR00-DR99]	40	0.20 (±0.60)	22.7
	Diseases of the eye and adnexa [DH00-DH59]	32	0.16 (±0.56)	18.2
	Diseases of the ear and mastoid process [DH60-DH95]	30	0.15 (±0.57)	17.0
	Diseases of the respiratory system [DJ00-DJ99]	26	0.13 (±0.50)	14.8
	Congenital malformations, deformations and chromosomal abnormalities [DQ00-DQ99]	15	0.08 (±0.39)	8.5
**Ilulissat**				
	Diseases of the eye and adnexa [DH00-DH59]	22	0.19 (±0.79)	22.9
	Diseases of the ear and mastoid process [DH60-DH95]	9	0.08 (±0.38)	9.4
	Diseases of the respiratory system [DJ00-DJ99]	8	0.07 (±0.26)	8.3
	Congenital malformations, deformations and chromosomal abnormalities [DQ00-DQ99]	7	0.06 (±0.34)	7.3
	Mental and behavioural disorders [DF00-DF99]	7	0.06 (±0.34)	7.3
	Symptoms, signs and abnormal clinical and laboratory findings, not elsewhere classified [DR00-DR99]	7	0.06 (±0.28)	7.3

N=number of contacts, SD=standard deviation.
Z-diagnoses excluded.

Supplementary Table 6: The five most frequent ICD-10 codes for contacts to the primary health care sector for boys/girls and Nuuk/Ilulissat.

	ICD-10 code	N	Mean (SD)	Incidence rate (1000/year)
**Male**				
	Conjunctivitis UNS (H109)	41	1.71 (±2.07)	197.4
	Otitis media UNS (H669)	40	1.67 (±2.32)	192.6
	Acute nasopharyngitis UNS (J009)	36	1.50 (±1.89)	173.3
	Cough UNS (R059)	30	1.25 (±1.39)	144.4
	Rash UNS (R219)	29	1.21 (±1.14)	139.6
**Female**				
	Conjunctivitis UNS (H109)	51	1.82 (±2.40)	220.6
	Acute nasopharyngitis UNS (J009)	51	1.82 (±1.83)	220.6
	Rash UNS (R219)	45	1.61 (±2.01)	194.6
	Fever UNS (R509)	39	1.39 (±1.85)	168.7
	Cough UNS (R059)	38	1.36 (±1.37)	164.3
**Nuuk**				
	Acute nasopharyngitis UNS (J009)	79	1.98 (±1.98)	234.4
	Conjunctivitis UNS (H109)	77	1.93 (±2.44)	228.5
	Cough UNS (R059)	65	1.63 (±1.39)	192.9
	Otitis media UNS (H669)	60	1.50 (±1.81)	178.0
	Rash UNS (R219)	58	1.45 (±1.74)	172.1
**Ilulissat**				
	Fever UNS (R509)	17	1.42 (±1.51)	166.8
	Rash UNS (R219)	16	1.33 (±1.44)	157.0
	Conjunctivitis UNS (H109)	15	1.25 (±1.29)	147.2
	Otitis media UNS (H669)	13	1.08 (±1.68)	127.6
	Otalgia (H920)	10	0.83 (±1.34)	98.1

N=number of contacts, SD=standard deviation. Study population = 52.
Z0-diagnoses and vaccinations excluded.

Supplementary Table 7: The five most frequent ICD-10 categories for contacts to the primary health care sector for boys/girls and Nuuk/Ilulissat.

	**ICD-10 category**	**N**	**Mean (SD)**	**Incidence rate (1000/year)**
Male				
	Symptoms, signs and abnormal clinical and laboratory findings, not elsewhere classified [DR00-DR99]	125	5.21 (±3.20)	601.8
	Diseases of the respiratory system [DJ00-DJ99]	119	4.96 (±4.21)	572.9
	Diseases of the ear and mastoid process [DH60-DH95]	78	3.25 (±3.31)	375.5
	Certain infectious and parasitic diseases [DA00-DB99]	68	2.83 (±2.46)	327.4
	Injury, poisoning and certain other consequences of external causes [DS00-DT98]	53	2.21 (±1.38)	255.2
**Female**				
	Symptoms, signs and abnormal clinical and laboratory findings, not elsewhere classified [DR00-DR99]	153	5.46 (±3.61)	661. 7
	Diseases of the respiratory system [DJ00-DJ99]	139	4.96 (±3.46)	601.1
	Certain infectious and parasitic diseases [DA00-DB99]	75	2.68 (±2.28)	324.4
	Diseases of the ear and mastoid process [DH60-DH95]	71	2.54 (±1.90)	307.1
	Diseases of the skin and subcutaneous tissue [DL00-DL99]	59	2.11 (±2.03)	255.2
**Nuuk**				
	Diseases of the respiratory system [DJ00-DJ99]	223	5.58 (±3.90)	661.7
	Symptoms, signs and abnormal clinical and laboratory findings, not elsewhere classified [DR00-DR99]	216	5.40 (±3.54)	640.9
	Diseases of the ear and mastoid process [DH60-DH95]	115	2.88 (±2.59)	341.2
	Certain infectious and parasitic diseases [DA00-DB99]	115	2.88 (±2.37)	341.2
	Diseases of the eye and adnexa [DH00-DH59]	87	2.18 (±2.47)	258.1
**Ilulissat**				
	Symptoms, signs and abnormal clinical and laboratory findings, not elsewhere classified [DR00-DR99]	62	5.17 (±2.98)	608.4
	Diseases of the respiratory system [DJ00-DJ99]	35	2.92 (±2.58)	343.5
	Diseases of the ear and mastoid process [DH60-DH95]	34	2.83 (±2.92)	333.7
	Certain infectious and parasitic diseases [DA00-DB99]	28	2.33 (±2.31)	274.8
	Diseases of the skin and subcutaneous tissue [DL00-DL99]	20	1.67 (±1.88)	196.3
	Injury, poisoning and certain other consequences of external causes [DS00-DT98]	20	1.67 (±1.07)	196.3

N=number of contacts, SD=standard deviation. Study population = 52.
Z-diagnoses excluded.
